# Association of atherogenic indices with myocardial damage and mortality in COVID-19

**DOI:** 10.1371/journal.pone.0302984

**Published:** 2024-05-16

**Authors:** Seyda Gunay-Polatkan, Serhat Caliskan, Deniz Sigirli

**Affiliations:** 1 Bursa Uludag University Faculty of Medicine, Department of Cardiology, Gorukle/Bursa, Turkey; 2 Istanbul Bahcelievler State Hospital Department of Cardiology, Bahcelievler/Istanbul, Turkey; 3 Bursa Uludag University Faculty of Medicine, Department of Biostatistics, Gorukle/Bursa, Turkey; Kerman University of Medical Sciences Physiology Research Center, ISLAMIC REPUBLIC OF IRAN

## Abstract

**Background:**

Lipoproteins in cell membranes are related to membrane stability and play a role against microorganisms. Patients with COVID-19 often experience myocyte membrane damage.

**Objective:**

This study aimed to search the relationship of atherogenic indices with myocardial damage and mortality in COVID-19.

**Methods:**

This was an observational, single-center, retrospective study. The study population was grouped according to in-hospital mortality. C-reactive protein (CRP), CRP to albumin ratio (CAR), monocyte to high density lipoprotein cholesterol ratio (MHR), levels of total cholesterol (TC), triglycerides, high-density lipoprotein cholesterol (HDLc), and low-density lipoprotein cholesterol (LDLc) and cardiac troponin I (cTnI) were recorded. Atherogenic indices (plasma atherogenic index [AIP], atherogenic coefficient [AC], Castelli’s risk indices I and II [CRI I and II], triglyceride to HDLc ratio (THR) were calculated.

**Results:**

A total of 783 patients were included. The mortality rate was 15.45% (n = 121). The median age of non-survivor group (NSG) was higher than survivor group (SG) [66.0 years (Q1 –Q3: 55.0–77.5) vs 54.0 years (Q1 –Q3: 43.0–63.0)] (p < 0.001). Study parameters which were measured significantly higher in the NSG were CRP, cTnI, triglyceride, CRI-I, CRI-II, AC, AIP, ferritin, CAR, MHR and THR. LDLc, HDLc, TC and albumin were significantly lower in NSG (p<0.001).

**Conclusion:**

THR is positively correlated with myocardial damage and strongly predicts in-hospital mortality in COVID-19.

## Introduction

Severe acute respiratory syndrome coronavirus-2 (SARS-CoV-2) has caused the pandemic that has challenged healthcare systems worldwide. Since 2019, there have been 770.778.396 confirmed cases of COVID-19, including 6.958.499 deaths, as of 21 September 2023, according to the World Health Organization (WHO) dashboard (https://covid19.who.int/). SARS-CoV-2 can infect the respiratory, cardiovascular, intestinal, hepatic and central neurological systems [1,2]. The severity of the disease is heterogeneous, and in some patients the infection can be fatal due to multiple organ failures [3]. Patients with COVID-19 may experience cardiovascular complications such as myocardial damage, arrhythmia, heart failure, or cardiac arrest [4–6]. As a biomarker of myocardial damage, cardiac troponin I levels have been reported to be elevated in approximately 5–25% of hospitalized COVID-19 patients [7,8]. SARS-CoV-2 infection triggers the production of many cytokines such as IL-1, IL-6, TNF-a, and IFN-gamma which causes the cytokine release syndrome leading to myocyte membrane destruction. In addition to the cytokine storm syndrome, both ischemic and non-ischemic mechanisms including hypoxia, sepsis, cardiac adrenergic hyperstimulation, pulmonary thromboembolism and myocarditis also cause myocardial damage [9]. At the time of the pandemic, there are so many patient applications for both diagnosis and post-discharge check-ups that the health system has difficulty in responding to this intensity. During this course it is important to predict the prognosis because prompt and precise treatment decisions can lower mortality. Numerous risk variables have been identified in earlier investigations for predicting COVID-19 mortality, but no clear consensus has been reached [10].

A crucial lipid component of cell membranes, cholesterol, ensures the normally functioning of the membrane by modulating membrane fluidity, integrity and segregation [11,12]. Hence, lipoproteins play a role in the membrane stability. The instability of myocyte cell membranes may lead to necrosis and myocardial damage. Lipoproteins also have a well-established immune function in the initial defense against microorganisms [13]. Bacterial, viral, and parasitic diseases may change lipoprotein levels [14,15]. Critically ill patients, particularly those with sepsis, have been documented to have hypolipidemia [16–19]. Hypolipidemia including lower levels of total cholesterol (TC), low density lipoprotein cholesterol (LDLc) and high density lipoprotein cholesterol (HDLc) develops in patients with modest symptoms during SARS-CoV-2 infection. In correlation with the severity of the disease, lipid levels continue to decrease with disease progression. On the other hand, a relationship between the severity of COVID-19 and high triglyceride (TG) levels was demonstrated [20–23].

In spite of a normal lipid profile, the possibility of coronary artery disease (CAD) cannot be ruled out. Hence, it has been suggested to use different combinations of these lipid profile parameters to identify high risk individuals. These lipid indices that predict prognosis in cardiovascular diseases include Castelli’s risk indices I and II, the plasma atherogenic index, non-HDLc and the atherogenic coefficient [24–27]. These calculated fractions can be used to assess the risk of cardiovascular disease beyond the routinely used lipid profile. For example, Bhardwaj et al. reported that even if TC and LDLc were not different between control group and patients with CAD, the ratios based on these parameters were significantly different [26]. Although previous studies investigated cholesterol and triglyceride levels in COVID-19 patients, to the best of our knowledge, these atherogenic indices have not been studied in relation to cardiovascular complications and mortality in COVID-19. This study aimed to search the relationship of atherogenic indices with myocardial damage and death in COVID-19.

## Materials and method

### Study design and study population

This was a retrospective, single-center, observational study of patients with COVID-19. Consecutive patients who applied to Republic of Turkey Ministry of Health, Istanbul Bahcelievler State Hospital between January 1, 2021 and December 1, 2021 were screened. A total of 783 patients who were tested with Bio-Speedy™ SARS-CoV-2 Double Gene™ RT-qPCR kit (Bioeksen, Istanbul, Türkiye) using specimens derived from nasopharyngeal swabs on admission and were diagnosed as confirmed case for COVID-19 were included. The patients who were not tested for lipid profile and cardiac troponin I (cTnI) on admission were not included. Also, the patients with a negative RT-PCR result were excluded. Patients who were not eligible for outpatient treatment according to The Republic of Türkiye Ministry of Health outpatient treatment criteria for COVID-19 [28] were all hospitalized and the study included only hospitalized patients, outpatient treatment group were excluded. Criteria for outpatient treatment eligibility are as follows:

The Republic of Türkiye Ministry of Health outpatient treatment criteria for COVID-19 [28]:

- Findings including fever, muscle/joint pains, cough, sore throat and nasal congestion with respiratory rate <30/minute, SpO2 level > 90% at room air;
- No underlying comorbid disease (cardiovascular diseases, diabetes mellitus, hypertension, cancer, chronic lung diseases, and other immunosuppressive conditions) and under the age of 50 years;
- No bad prognostic measures in blood examinations upon admission (blood lymphocyte count <800/μl or C-reactive protein (CRP) >40 mg/L or ferritin >500ng/ml or D-Dimer >1000 ng/ml); and
- Mild pneumonia findings in lung X-ray or chest computerized tomography

Patients were classified into two groups according to in-hospital mortality: a survivor group (SG) and a non-survivor group (NSG). SG was defined as the patients who recovered. Demographic characteristics and results of laboratory tests on admission were retrospectively collected from medical records. Clinical outcome (death or recovery) and presence of myocardial damage were the primary outcomes. Recovery was described as relief of fever and other symptoms, resolution of lung infiltration and 2 negative RT-PCR test results. According to the previous studies, myocardial damage was defined if the serum levels of cardiac biomarkers (eg cardiac troponin I) were above the 99th percentile upper reference limit [7,8]. In this study, myocardial damage was defined as blood levels of cTnI were above 17.5 pg/mL which is the 99th-percentile upper reference limit of study kit.

This study was conducted in accordance with the declaration of Helsinki [29] and was approved by the Turkish Ministry of Health and the local institutional review board (Istanbul Bakirkoy Dr. Sadi Konuk Education and Research Hospital) (approval date:18.10.2021, approval no:2021-20-21). Data were accessed for research purposes after ethics committee had approved the research between October 18, 2021 and December 1, 2021). All data were fully anonymized before access and ethics committee waived the requirement for written informed consent since it was designed as retrospectively.

### Data collection

The demographic characteristics (age, gender and comorbidities), laboratory findings on admission (complete blood count and biochemical tests) and outcome data were collected from patient files.

The hematological parameters were determined using XT-4000i Hematology Analyzer (Sysmex, Kobe, Japan). Roche Cobas e411 analyzer (Hitachi High-Technologies Corporation, Tokyo, Japan) was used to measure cTnI. The AU5800 Clinical Chemistry System (Beckman Coulter, Inc. California, USA) was used to measure CRP, albumin and lipid profile (TC, HDLc, LDLc, and TG levels).

Using these results MHR and CAR as inflammation-based prognostic parameters were calculated in accordance with previous studies [30,31]. The indices used in this study were calculated as follows: Castelli’s risk indices I (CRI-I) = (TC/HDLc) [23], Castelli’s risk indices II (CRI-II) = (LDLc/HDLc) [23], plasma atherogenic index (AIP) = (log TG/HDLc) [24], non-HDLc = (TC–HDLc) [25], and atherogenic coefficient (AC) = (non-HDLc/HDLc) [26].

### Statistical analyses

Normality of the data were tested with the Shapiro-Wilk test. For variables that did not follow normal distribution, the Mann-Whitney U test was used to compare two independent groups and median (1st quartile-3rd quartile) values were given. Counts and percentages were used to express categorical variables and Pearson chi-square test was used to analyze them. The correlations between the variables were examined with Spearman’s correlation coefficient. Receiver operating characteristic (ROC) analysis was performed to evaluate the performance of some laboratory variables and indices in distinguishing between survivors and non-survivors. We performed logistic regression (LR) analysis to determine risk factors for in-hospital mortality. We conducted stepwise backwards LR routine, by taking variables that were found statistically significant in univariate analysis. We excluded variables which are mathematical functions of the other variables and cause inflated odds ratios. We dichotomized the baseline laboratory variables, by using their optimal cut-off values obtained from ROC curve analysis. Level of significance was α = 0.05. IBM SPSS Statistics version 28.0 (IBM Corp., NY, USA) and MedCalc® version 12.3.0.0 were used for the statistical analyses.

## Results

### Patients characteristics

A total of 783 patients were included. All patients had positive RT-PCR test results for COVID-19. The rate of mortality was 15.45% (n = 121). There were 63 male patients (52.1%) in NSG and 314 male patients (47.4%) in SG (p = 0.35). The median age of NSG was higher than SG [66.0 years (Q1 –Q3: 55.0–77.5) vs 54.0 years (Q1 –Q3: 43.0–63.0)] (p < 0.001). The median time in-hospital was 12.0 days (Q1- Q3: 8.0–16.0) in NSG and 5.0 days (Q1- Q3: 5.0–7.0) in SG (p < 0.001). Diabetes mellitus, hypertension, hyperlipidemia and renal failure were more common in NSG than SG (p < 0.001) (Table 1).

**Table 1:** Baseline clinical characteristics of the study groups.

Variables	Survivor group n = 662	Non-survivor group n = 121	*p*-value
Age (years)*	54.0 (43.0–63.0)	66.0 (55.0–77.5)	<0.001
Gender^#^ Male	314 (47.4)	63 (52.1)	0.348
Female	348 (52.6)	58 (47.9)	
In-hospital time (days)*	5.0 (5.0–7.0)	12.0 (8.0–16.0)	<0.001
Diabetes mellitus^#^	125 (18.9)	42 (34.7)	<0.001
Hypertension^#^	218 (32.9)	81 (66.9)	<0.001
Hyperlipidemia^#^	14 (2.1)	21 (17.4)	<0.001
CAD^#^	59 (8.9)	30 (24.8)	<0.001
Renal failure^#^	27 (4.1)	20 (16.5)	<0.001

CAD: Coronary artery disease.

Data presented with

*the median (Q1-Q3) or

^#^ n(%) values.

Laboratory test results showed that CRP, cTnI, triglyceride, CRI-I, CRI-II, AC, AIP, ferritin, CAR, MHR and THR were significantly higher in the NSG than those in the SG, while LDLc, HDLc, TC and albumin were significantly lower in NSG than SG (Table 2).

**Table 2:** Baseline laboratory parameters of the study groups.

Variables	Survivor group n = 662	Non-survivor group n = 121	*p*-value
LDLc (mg/dl)	91.00 (71.75–114.00)	75.00 (59.00–102.50)	<0.001
HDLc (mg/dl)	43.00 (37.00–50.00)	33.00 (28.00–38.50)	<0.001
TC (mg/dl)	160.00 (134.00–184.25)	143.00 (122.00–175.50)	0.003
TG (mg/dl)	106.00 (85.00–126.00)	169.00 (129.50–218.50)	<0.001
Non-HDLc (mg/dl)	113.00 (92.00–136.25)	109.00 (89.00–141.00)	0.717
CRI-I	3.65 (3.13–4.22)	4.53 (3.84–5.39)	<0.001
CRI-II	2.12 (1.67–2.69)	2.44 (1.71–3.03)	0.007
AC	2.65 (2.13–3.22)	3.53 (2.84–4.39)	<0.001
AIP	0.04 ((-0.07)-0.12)	0.34 (0.23–0.46)	<0.001
CRP (mg/L)	30.20 (14.00–59.20)	153.00 (92.45–234.00)	<0.001
cTnI (pg/mL)	3.77 (2.07–7.16)	55.20 (22.13–126.00)	<0.001
AST (IU/L)	25.00 (19.00–37.00)	47.00 (28.50–66.00)	<0.001
ALT (IU/L)	26.00 (16.00–44.00)	37.00 (19.50–55.50)	0.001
Ferritin (ng/mL)	211.00 (108.00–399.50)	655.00 (380.00–923.00)	<0.001
Albumin (g/L)	39.20 (37.10–41.50)	29.80 (27.00–33.10)	<0.001
Creatinine (mg/dl)	0.75 (0.62–0.89)	0.86 (0.69–1.13)	<0.001
Hemoglobin (g/dL)	12.70 (11.70–13.80)	11.80 (10.90–13.30)	<0.001
WBC (10^3^/mm^3^)	6.81 (5.00–9.14)	9.64 (6.73–13.97)	<0.001
Platelets (10^3^/mm^3^)	241.00 (193.00–319.00)	211.00 (173.50–288.00)	0.001
Neutrophils (10^3^/mL)	5.30 (3.58–7.60)	8.02 (5.84–13.28)	<0.001
Lymphocytes (10^3^/mL)	1.01 (0.69–1.38)	0.62 (0.45–0.94)	<0.001
Monocytes (10^3^/mL)	0.34 (0.22–0.51)	0.38 (0.23–0.66)	0.064
CAR	0.77 (0.34–1.54)	5.28 (2.79–7.70)	<0.001
MHR	0.008 (0.005–0.012)	0.012 (0.006–0.021)	<0.001
THR	2.53 (1.93–3.05)	5.00 (3.93–6.61)	<0.001

Data given as; median (Q1-Q3).

AC: Atherogenic coefficient; AIP: Atherogenic index of plasma; CRI-I: Castelli’s risk index I; CRI-II: Castelli’s risk index II; HDLc: High-density lipoprotein cholesterol; LDLc: Low-density lipoprotein cholesterol; TC: Total cholesterol; TG: Triglyceride; cTnI: Cardiac troponin I; CRP: C-reactive protein; CAR: CRP/albumin; MHR: Monocyte/HDL; THR: Triglyceride/HDL; ALT: Alanine aminotransferase; AST: Aspartate aminotransferase; WBC: White blood cell.

cTnI levels of 128 patients (16.3%) were more than 17.5 pg/mL (upper normal limit of cTnI test kit). Of these, 33 patients (5%) were in SG and 95 patients (78.5%) were in NSG (p<0.001). When cTnI and inflammation markers were correlated with the lipid profile and atherogenic indices, it was found that LDLc, HDLc and TC were negatively correlated with CRP, CAR and cTnI. CRI-I, AC, AIP, triglyceride and THR were positively correlated with CRP, CAR, MHR and cTnI (p < 0.001 for all) (Table 3).

**Table 3:** Correlations of the atherogenic indices and lipid profile levels with inflammation and myocardial damage in COVID-19 patients.

	CRP		CAR		MHR		cTnI	
	r	p-value	r	p-value	R	p-value	r	p-value
HDLc	-0.261	<0.001*	-0.265	<0.001*	-0.400	<0.001*	-0.196	<0.001*
TG	0.372	<0.001*	0.413	<0.001*	0.093	0.009*	0.182	<0.001*
CRI-I	0.211	<0.001*	0.219	<0.001*	0.325	<0.001*	0.156	<0.001*
AC	0.211	<0.001*	0.219	<0.001*	0.325	<0.001*	0.156	<0.001*
AIP	0.397	<0.001*	0.426	<0.001*	0.295	<0.001*	0.256	<0.001*
THR	0.274	<0.001*	0.291	<0.001*	0.215	<0.001*	0.254	<0.001*

r: Spearman correlation coefficient, AC: Atherogenic coefficient; AIP: Atherogenic index of plasma; CRI-I: Castelli’s risk index I; CRI-II: Castelli’s risk index II; HDLc: High-density lipoprotein cholesterol; LDLc: Low-density lipoprotein cholesterol; TC: Total cholesterol; TG: Triglyceride; THR: Triglyceride/HDL; cTnI: Cardiac troponin I; CRP: C-reactive protein; CAR: CRP/albumin; MHR: Monocyte/HDL

### Association between clinical outcomes and baseline characteristics

**Fig 1:**
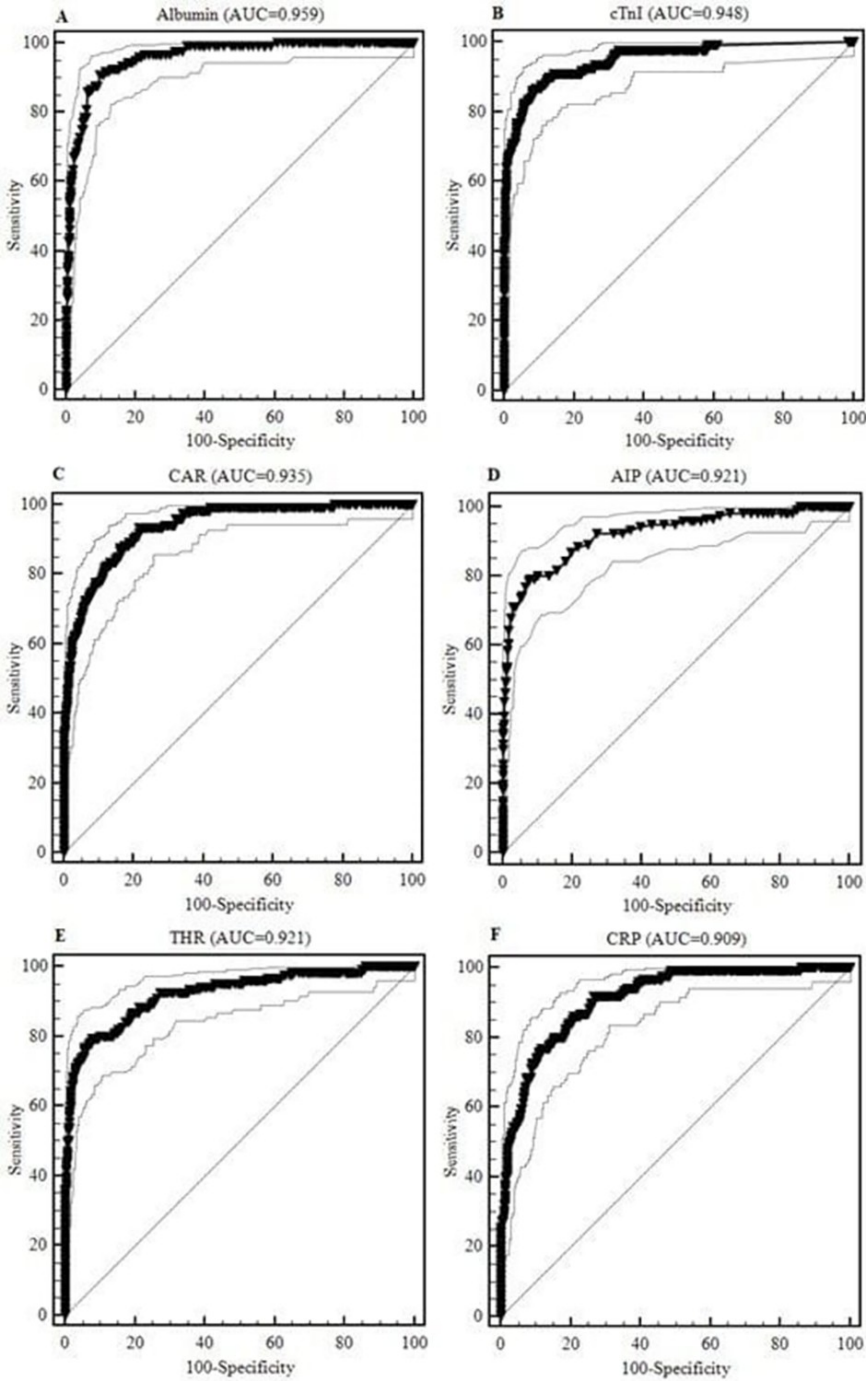
ROC curves for A) Albumin, B) cTnI, C) CAR, D) AIP, E) THR and F) CRP. AIP: Atherogenic index of plasma; CAR: CRP/albumin THR: Triglyceride/HDLc; CRP: C-reactive protein; cTnI: Cardiac troponin I.

Prognostic performance of laboratory findings including lipid profile parameters and cTnI as myocardial injury biomarker were evaluated using ROC curve analyses for predicting in-hospital mortality of patients with COVID-19. The area under the ROC curve (AUC) of the variables ranged between 0.577 and 0.959 with the highest AUC values for albumin, cTnI, CAR, AIP, THR and CRP (0.959, 0.948, 0.935, 0.921,0.921 and 0.909, respectively) (Fig 1).

The optimal cut-off values were calculated, which corresponded to ≤ 35 g/L for albumin, >13.63 pg/mL for cTnI, >1.68 for CAR, >0.2 for AIP, >3.68 for THR and >90.6 mg/L for CRP (Table 4).

**Table 4:** Results of the ROC curve analysis.

Variables	AUC	p-value	SE of AUC	Youden J	cut-off value	Sensitivity (95% CI)	Specificity (95% CI)	PPV (95% CI)	NPV (95% CI)
Albumin	0.959	<0.001	0.008	0.811	≤35	90.91 (84.3 - 95.4)	90.18 (87.7 - 92.3)	62.90 (55.2 - 70.0)	98.20 (96.8 - 99.1)
cTnI	0.948	<0.001	0.012	0.778	>13.63	85.95 (78.5 - 91.6)	91.84 (89.5 - 93.8)	65.80 (57.8 - 73.2)	97.30 (95.7 - 98.4)
CAR	0.935	<0.001	0.011	0.718	>1.68	93.39 (87.4 - 97.1)	78.40 (75.1 - 81.5)	44.10 (38.0 - 50.5)	98.50 (97.0 - 99.3)
AIP	0.921	<0.001	0.015	0.713	>0.2	79.34 (71.0 - 86.2)	91.99 (89.7 - 93.9)	64.40 (56.2 - 72.1)	96.10 (94.2 - 97.4)
THR	0.921	<0.001	0.016	0.715	>3.68	79.34 (71.0 - 86.2)	92.15 (89.8 - 94.1)	64.90 (56.6 - 72.5)	96.10 (94.2 - 97.4)
CRP	0.909	<0.001	0.014	0.652	>90.6	76.86 (68.3 - 84.0)	88.37 (85.7 - 90.7)	54.70 (46.9 - 62.4)	95.40 (93.5 - 96.9)
TG	0.843	<0.001	0.022	0.582	>139	72.73 (63.9 - 80.4)	85.50 (82.6 - 88.1)	47.80 (40.4 - 55.3)	94.50 (92.3 - 96.2)
In-hospital time	0.840	<0.001	0.026	0.613	>8	72.73 (63.9 - 80.4)	88.52 (85.8 - 90.8)	53.70 (45.7 - 61.5)	94.70 (92.6 - 96.3)
Ferritin	0.829	<0.001	0.021	0.516	>311	86.55 (79.1 - 92.1)	65.05 (61.3 - 68.7)	30.80 (25.9 - 36.1)	96.40 (94.2 - 97.9)
HDLc	0.786	<0.001	0.024	0.455	≤37	72.73 (63.9 - 80.4)	72.81 (69.2 - 76.2)	32.80 (27.2 - 38.8)	93.60 (91.1 - 95.5)
Neutrophils	0.722	<0.001	0.027	0.373	>6.35	73.55 (64.8 - 81.2)	63.75 (60.0 - 67.4)	27.10 (22.3 - 32.2)	93.00 (90.2 - 95.1)
Lymphocytes	0.721	<0.001	0.025	0.332	≤0.64	53.72 (44.4 - 62.8)	79.46 (76.2 - 82.5)	32.30 (25.9 - 39.3)	90.40 (87.7 - 92.6)
AC	0.711	<0.001	0.028	0.353	>3.31	57.85 (48.5 - 66.8)	77.49 (74.1 - 80.6)	32.00 (25.8 - 38.6)	91.00 (88.3 - 93.2)
CRI-I	0.711	<0.001	0.028	0.353	>4.31	57.85 (48.5 - 66.8)	77.49 (74.1 - 80.6)	32.0 (25.8 - 38.6)	91.00 (88.3 - 93.2)
WBC	0.693	<0.001	0.028	0.305	>9.37	53.72 (44.4 - 62.8)	76.74 (73.3 - 79.9)	29.70 (23.7 - 36.2)	90.10 (87.3 - 92.4)
MHR	0.644	<0.001	0.030	0.253	>0.0134	44.63 (35.6 - 53.9)	80.66 (77.4 - 83.6)	29.70 (23.1 - 36.9)	88.90 (86.1 - 91.3)
LDLc	0.629	<0.001	0.029	0.237	≤78	55.37 (46.1 - 64.4)	68.28 (64.6 - 71.8)	24.20 (19.3 - 29.7)	89.30 (86.3 - 91.9)
Hemoglobin	0.625	<0.001	0.029	0.236	≤11.5	46.28 (37.2 - 55.6)	77.34 (74.0 - 80.5)	27.20 (21.2 - 33.8)	88.70 (85.9 - 91.2)
Creatinine	0.621	<0.001	0.031	0.224	>0.88	47.93 (38.8 - 57.2)	74.47 (71.0 - 77.8)	25.60 (20.0 - 31.7)	88.70 (85.7 - 91.2)
Platelets	0.594	0.001	0.028	0.180	≤220	56.20 (46.9 - 65.2)	61.78 (58.0 - 65.5)	21.20 (16.8 - 26.1)	88.50 (85.3 - 91.3)
TC	0.585	0.004	0.029	0.179	≤143	52.07 (42.8 - 61.2)	65.86 (62.1 - 69.5)	21.80 (17.2 - 27.0)	88.30 (85.1 - 91.0)
CRI-II	0.577	0.012	0.031	0.173	>2.39	53.72 (44.4 - 62.8)	63.60 (59.8 - 67.3)	21.20 (16.8 - 26.3)	88.30 (85.0 - 91.0)
Monocytes	-	0.089	-	-	-	-	-	-	-
Non-HDLc	-	0.726	-	-	-	-	-	-	-

AUC: Area under the ROC curve, CI: confidence interval, SE: standard error, PPV: positive predictive value, NPV: negative predictive value

Variables are sorted in the table according to AUC values.

AC: Atherogenic coefficient; AIP: Atherogenic index of plasma; CRI-I: Castelli’s risk index I; CRI-II: Castelli’s risk index II; HDLc: High-density lipoprotein cholesterol; LDLc: Low-density lipoprotein cholesterol; TC: Total cholesterol; TG: Triglyceride; THR: Triglyceride/HDL; cTnI: Cardiac troponin I; CRP: C-reactive protein; CAR: CRP/albumin; MHR: monocyte/HDL.

We performed stepwise backwards logistic regression analysis by including the following variables: categorized albumin, cTnI, AIP, THR, CRP, ferritin, neutrophils, lymphocytes, AC, CRI-1, white blood cell, MHR, hemoglobin, platelets, TC and creatinine. At the last model (p-value for the Omnibus test of model coefficients: p<0.001, -2 log-likelihood value: 90.15, Cox&Snell R^2^ = 0.52, Nagelkerke R^2^ = 0.921, p-value for the Hosmer-Lemeshow test: p = 0.983, overall correct classification percentage of the model: 98.33%), categorized albumin, cTnI, THR, CRP, lymphocytes, AC and TC were found to be significant risk factors in predicting in-hospital mortality. THR levels being >3.68, increased the risk of mortality 112.57 times (p<0.001). Odds ratio of THR was the highest odds ratio value among study variables (Table 5). Variance inflation factors for the independent variables were less than 2, changing from 1.091 to 1.775. ROC curve analysis were performed and AUC calculated for the model predicted probabilities to evaluate the performance of the model. AUC was estimated to be 0.996 (p<0.001), cut-off value was 0.168, sensitivity, specificity, positive and negative predictive values found to be 96.64, 96.67, 83.90 and 99.40, respectively.

**Table 5:** Results of the stepwise backwards logistic regression analysis (last model).

Variables	p-value	OR	95% CI for OR	
Albumin (RC: ≤35)	<0.001	19.85	5.47	72.04
cTnI (RC: >13.63)	<0.001	66.17	14.08	310.96
THR (RC: >3.68)	<0.001	112.57	18.38	689.50
CRP (RC: >90.6)	<0.001	55.15	11.90	255.59
Ferritin (RC: >311)	0.092	3.25	0.82	12.86
Lymphocytes (RC: ≤0.64)	<0.020	5.03	1.30	19.54
AC (RC: >3.31)	0.090	3.37	0.83	13.74
TC (RC: ≤143)	<0.002	9.91	2.30	42.75

RC: Reference category; OR: Odds ratio; CI: Confidence interval; cTnI: Cardiac troponin I; THR: Triglyceride/HDL; CRP: C-reactive protein; AC: Atherogenic coefficient TC: Total cholesterol.

## Discussion

In this study, we found that THR levels more than 3.68 increased the risk of mortality and the odds ratio of THR was the highest odds ratio value among study variables. Also, in consistence with previous studies, CRP and cTnI values were higher in NSG [32–34]. Among the study parameters; LDLc, HDLc and TC were negatively correlated with markers of myocardial damage (cTnI) and inflammation (CRP, CAR and MHR), while triglyceride and atherogenic indices (CRI-I, AC, AIP and THR) were positively correlated with them. Triglyceride was positively and HDLc was negatively correlated with myocardial damage. Since THR is calculated by dividing the triglyceride levels by the HDLc levels, an increase in triglyceride level and decrease in HDLc level is expected to give a higher THR value. In our study, in consistence with this calculation THR showed a positive correlation with myocardial damage.

The protective effect of HDLc against atherosclerosis was reported in various studies [35,36]. Besides atherosclerosis, HDLc also decreases myocardial damage. Theilmeier et al. reported that when used in a mouse model of cardiac ischemia/reperfusion, HDLc also decreased cardiomyocyte apoptosis [37]. In severely ill COVID-19 patients, another cause of myocardial damage is abnormal thrombosis. According to epidemiological studies, the risk of thrombosis and HDLc levels reported to be inversely correlated [38]. The negative correlation between HDLc and myocardial damage in our study is consistent with the results of these previous studies.

While patients with COVID-19 experience a cytokine storm; TC, HDLc, ApoA1, albumin, total protein levels decrease as well as CD16+ T, CD3+ T, and CD8+ T cell counts [20,39]. HDLc may have an antiviral effect, since RNA and DNA viruses may be covered by HDLc whether or not they are enclosed [14]. Hence, inflammatory responses against viral particles may also be affected by HDLc. Microarray tests of in vitro trials demonstrate that HDLc suppresses the lipopolysaccharide -stimulated macrophage genes that control the type I interferon response [40]. Also, acute inflammation can cause structural and functional modifications of HDLc, which might lead dysfunction in HDL particles [14]. In a previous study, only the most severe COVID-19 patients showed a substantial decline in HDLc, but all patient groups showed significant declines in TC and LDLc. Accordingly, it appears that hypolipidemia may develop in those who have minor symptoms and HDLc level decreases as the disease progresses [20]. As a result, viral infections may change the host’s lipid profile and severely ill patients with COVID-19 show lower levels of TC, HDLc and LDLc. Data about viral load was not present in our study. Because of that it is not clear whether the HDLc level was lower in severe patients because the viral load was higher, or whether the HDLc level was lower because inflammation was more severe. Although many previous studies aimed to determine disease severity using lipid levels in patients with COVID-19 [20], there are still very few studies investigating the role of atherogenic indices in myocardial damage and mortality.

In conclusion, although AIP and THR had the highest two AUCs among the atherogenic indices, THR had the highest odds ratio. Because THR is easier to calculate, it may be useful for predicting prognosis in patients with COVID-19. Further studies, including the relationship between viral load and lipid levels are also needed.

Study Limitations: This study did have some limitations. The first issue was the small sample size. Additionally, study population included only Turkish patients and so the results may not represent all patients from other nations or races. Another limitation was the study design. This was not a prospectively designed study and as a result follow-up data of SG after discharge was lack. Finally, any data about the viral load of patients was not present.

## Supporting information

S1 Data (XLSX)
